# Federated learning for lesion segmentation in multiple sclerosis: a real-world multi-center feasibility study

**DOI:** 10.3389/fneur.2025.1620469

**Published:** 2025-09-10

**Authors:** Sarah Hindawi, Bartlomiej Szubstarski, Eric Boernert, Björn Tackenberg, Jens Wuerfel

**Affiliations:** ^1^Hoffmann-La Roche Limited, Mississauga, ON, Canada; ^2^Roche Polska Sp. z o.o., Warsaw, Poland; ^3^F. Hoffmann-La Roche AG, Basel, Switzerland

**Keywords:** federated learning, MRI lesion segmentation, privacy-preserving AI, distributed deep learning, multi-site training

## Abstract

Multiple sclerosis (MS) is a chronic neuroinflammatory disease driven by immune-mediated central nervous system damage, often leading to progressive disability. Accurate segmentation of MS lesions on MRI is crucial for monitoring disease and treatment efficacy; however, manual segmentation remains time-consuming and prone to variability. While deep learning has advanced automated segmentation, robust performance benefits from large-scale, diverse datasets, yet data pooling is restricted by privacy regulations and clinical performance remains challenged by inter-site heterogeneity. In this proof-of-concept work, we aim to apply and adopt Federated Learning (FL) in a real-world hospital setting. We assessed FL for MS lesion segmentation using the self-configuring nnU-Net model, leveraging 512 MRI cases from three sites without sharing raw patient data. The federated model achieved Dice scores ranging from 0.66 to 0.80 across held-out test sets. While performance varied across sites, reflecting data heterogeneity, the study demonstrates the potential of FL as a scalable and secure paradigm for advancing automated MS analysis in distributed clinical environments. This work supports adopting secure, collaborative AI in neuroimaging, offering utility for privacy-sensitive clinical research and a starting point for medical AI development, bridging the gap between model generalizability and regulatory compliance.

## 1 Introduction

Multiple sclerosis is a chronic autoimmune disorder of the central nervous system (CNS) and is a leading cause of non-traumatic neurological disability among young adults (1). MS affects more than 2.8 million individuals worldwide (2). The disease is characterized by inflammatory demyelinating CNS lesions (3), which appear as hyperintense areas in white matter on T2-weighted/FLAIR MRI and are crucial for diagnosis and monitoring disease progression. Lesion burden correlates with disability (4), making accurate lesion segmentation vital for evaluating treatment efficacy.

Manual MS lesion segmentation is the clinical gold standard but is labor-intensive and prone to observer variability. Recent convolutional neural network approaches, including 3D U-Net variants, have achieved Dice scores of 0.6–0.8 for automated MS lesion segmentation on benchmark datasets (5). We employed nnU-Net, a self-configuring framework with strong performance across diverse medical segmentation tasks (6). Clinical adoption of automated segmentation methods remains limited due to the heterogeneity of MRI data, including variations in acquisition protocols, scanner types, and lesion characteristics across patient populations. Models trained on single-center data may generalize poorly to external data due to distribution shifts (7). Privacy regulations limit data sharing across centers, limiting the ability to curate sufficiently large and diverse training datasets. This fragmentation of data impedes the development of generalizable AI models and continues to hinder machine learning (ML) translation into clinical settings (8).

Federated learning (FL) has emerged as a promising solution by enabling collaborative model training without exchanging raw data. In a federated learning paradigm, each institution (client) trains a local copy of the global model on-site. Instead of transferring patient data, only the model’s learned parameters (e.g., weight updates) are shared with a central server. The server aggregates the updates from the participating clients to construct a consensus global model, enabling collaborative learning while addressing privacy concerns and utilizing otherwise inaccessible datasets. Despite its promise, FL is still in the early stages of medical deployment (9) Two studies from the same research group have investigated FL for MS lesion segmentation. These studies used simulated FL environments with clinical and public datasets (fewer than 200 subjects across scenarios) and reported moderate Dice scores ranging from 54 to 77% (10, 11). A recent study (12) also investigated FL for MS lesion segmentation as part of a broader benchmark of five neuroimaging tasks, conducted in a simulated FL environment, reporting Dice scores ranging from 63.2 to 70.2% on MSSEG dataset (13).

In contrast, our study deploys a federated learning framework for MS lesion segmentation in a real-world, multi-institutional setting, addressing legal and regulatory constraints that often hinder clinical translation. These challenges, typically underexplored in simulated environments, are addressed through a secure, end-to-end deployment in which each site retains full ownership and control of its data, demonstrating the practical feasibility of integrating FL into clinical practice under strict data governance. We trained and evaluated the model across three clinical institutions on a total of 512 MRI cases, integrating both academic research and routine clinical data. Specifically, we aim to establish a federated architecture for distributed image analysis and assess the feasibility of training a model for segmenting T2-weighted hyperintense MS lesions across sites. By demonstrating FL’s application to MS lesion segmentation, we aim to strengthen the groundwork for privacy-preserving, collaborative AI in neuroimaging.

## 2 Methods

### 2.1 Federated framework architecture

To enable privacy-preserving, multi-center training for MS lesion segmentation, we extended our federated learning platform with imaging capabilities by integrating it with an established open-source framework for radiology image processing. Specifically, we utilized Kaapana, an open-source platform described in (14, 15), to coordinate local imaging processing and computational workflows. Kaapana is a modular toolkit for medical image analysis that enables decentralized data access, data management, and remote execution of containerized algorithms. It supports private cloud development and integrates seamlessly with local clinical IT infrastructure. The platform employed a client–server FL architecture to train the model across three participating sites. Each client maintained a local copy of the model and trained it on its own dataset of MR images. A central server acted as the coordinating node, aggregating received model parameters using the Federated Averaging (FedAvg) algorithm, which computes a weighted average of the clients’ model weights (16). To address operational, security and collaboration network scalability needs in real-world clinical environments, we extended our setup with additional enterprise-grade computational governance capabilities developed by Apheris, enabling institutions to collaborate securely on distributed data within a governed and privacy-preserving framework. This integration allowed all collaborating institutions to retain end-to-end control over algorithm execution. Although open-source solutions offer transparency and adaptability, their integration into clinical workflows can introduce operational overhead, including the need for manual code reviews. To mitigate this challenge and reduce risk, we implemented a centralized algorithm review process with a controlled algorithm pull mechanism from a central container registry, ensuring reproducibility, data and model governance, and streamlined collaboration without exposing sensitive data.

By design, the federated model should be exposed to a wider variety of imaging patterns (patient demographics, scanner types, artifact profiles) than any single-site model, ideally resulting in a more generalizable model. MRI data were preprocessed using a standardized pipeline applied consistently across all sites to ensure uniform orientation and registration. We employed nnU-Net, which automatically configures its architecture, preprocessing, and training pipelines to the given dataset, enabling site-specific adaptation and efficient deployment with minimal computational and implementation overhead (6). The model was trained across sites using a uniform configuration and shared hyperparameters. Each site used locally managed infrastructure, typically comprising GPUs with at least 24 GB of VRAM (NVIDIA Turing or newer) and at least 64 GB of RAM. The training was done in a synchronous federated manner such that all sites participated in each round. By the end of training, the final federated model was evaluated on held-out test sets at each participating site.

Throughout the federated training, no MR images or patient identifiers were ever exchanged. Only data fingerprints, containing image sizes, voxel spacings, and intensity characteristics for model initialization, along with model parameters were shared during FL iterations. Dataset fingerprints were required for the adaptive, rule-based configuration of the segmentation pipeline, including the selection of the patch size, network topology, and batch size, all of which depend on image properties (6). This approach together with a decentralized architecture inherently preserves data privacy, as an adversary cannot directly access the underlying images through the central server. To further secure communications, all network traffic between the server and client nodes was encrypted using state-of-the-art protocols. Each participating site deployed and operated a local platform within its own firewall, allowing the central orchestration server to invoke nnU-Net federated training workflows on local data. We implemented local basic authentication for the nodes and an external identity and access mechanism for the central node. This design enables more autonomous, isolated and efficient deployment at each site. Node authentication within the federated network is based on a centrally generated token that each site receives via independent media during registration. This token includes: 1. an SSL certificate, 2. an authentication token, 3. connection details of the central instance and 4. a symmetrical encryption key as an additional protection mechanism for data in transit. To simplify deployment and avoid dependencies on potential vulnerabilities in the open-source network stack, we opted not to implement a dedicated virtual network infrastructure for federated nodes. Instead, we introduced a symmetric encryption layer implemented explicitly at the federated client and server applications. Due to its inherent speed, this mechanism was well-suited for encrypting client-generated weights at each round. Additionally, it served as a safeguard to ensure secure communication between clients and the central node, effectively replicating the protection typically provided by a virtual private network.

This setup guarantees the authenticity of the contributing clients and prevents spoofing or tampering within the federated network. Each client application maintained a list of approved datasets and workflows for federated processing, allowing site personnel to contribute to model training without relinquishing control over their data. This approach is compliant with data protection regulations and addresses the ethical concerns of data sharing.

**Figure 1:**
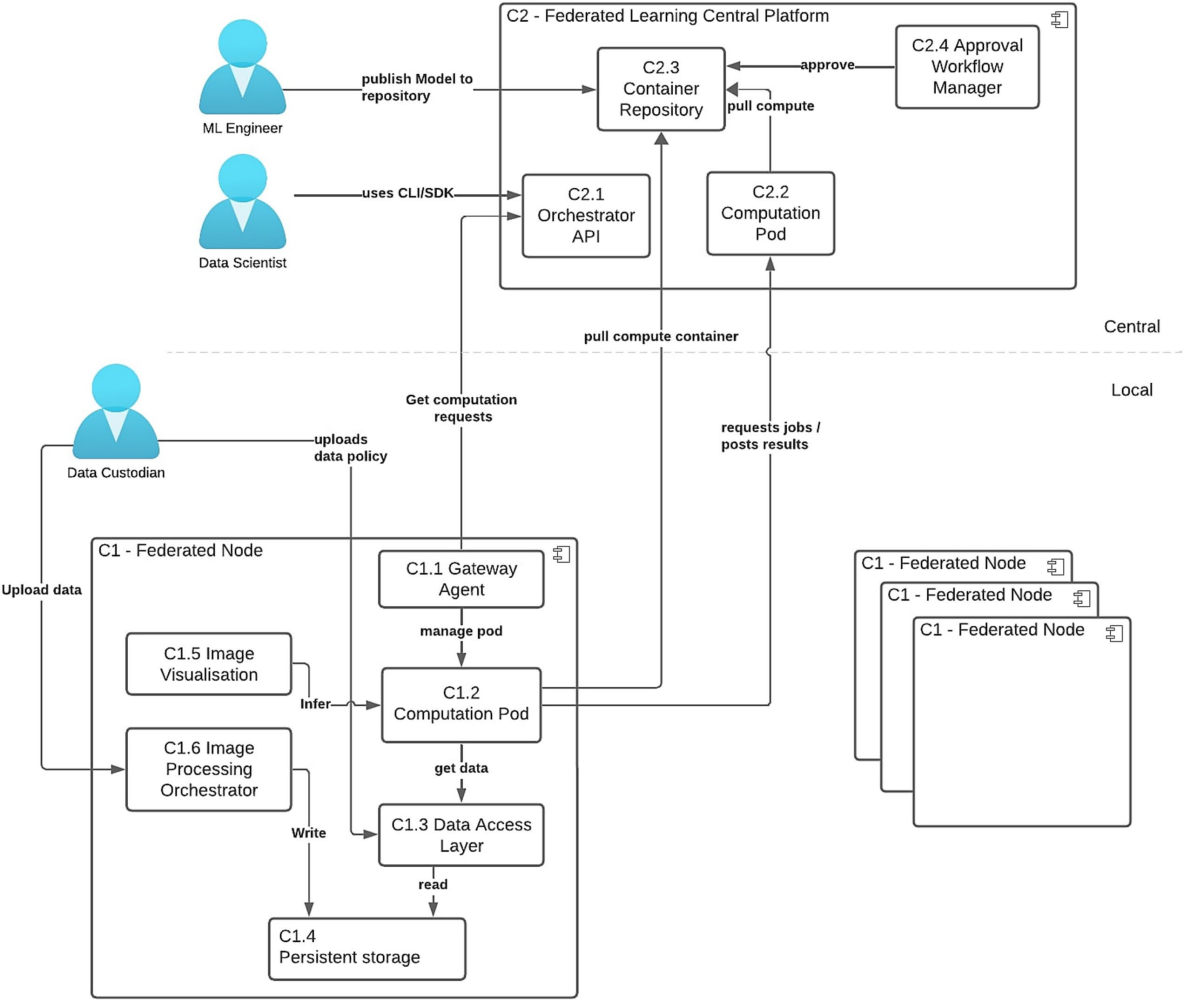
High-level solution architecture showing the integration of Kaapana and Apheris into a single solution. This extended architecture allows site personnel to control processing pipelines executed on their data. It supports three primary user workflows and illustrates basic component interactions at a single federated node.

The federated learning architecture (illustrated in Figure 1) supports the following key user workflows:

1. Model publication by ML Engineer - A locally tested model is converted into its federated version and uploaded to a central model repository. Once approved by the site Data Custodian, it becomes available for execution at the corresponding sites.
2. Upload of data assets and data access policies by the Data Custodian - At each site, the Data Custodian defines which models are authorized to access the uploaded data. This enables Gateway agents to accept requests to execute approved ML models.
3. Federated Workflow Execution by Data Scientist - Using the Python SDK, the Data Scientist interacts with the Federated Learning Orchestrator to initiate computation pods at the federated nodes (workers) and the central platform (aggregator), in accordance with the approved data access policies. This setup facilitates the full execution of nnU-Net training across the participating sites.

### 2.2 Description of datasets

This is a multi-center, multi-country study utilizing anonymized MRI data from patients with MS. The study involved in-house data from a previous Roche-sponsored trial at our site (Site A, 149 cases, each consisting of paired T1w and FLAIR images), as well as anonymized observational data from two academic medical centers: one in Switzerland (Site B, 325 cases) and one in Germany (Site C, 38 cases). A total of 512 expert-annotated MRI cases were used, of which 380 were allocated to training and validation. Patients were uniquely assigned to either the training/validation or test sets to avoid data leakage and ensure unbiased model evaluation. In accordance with data protection principles, all data remained local at each site and were never shared centrally.

The datasets included 1 mm isotropic 3D T2-weighted/FLAIR and T1-weighted sequences (with a tolerance of ±0.1 mm, ranging from 0.9 to 1.1 mm). Scans were acquired on Siemens, Philips, and GE Medical Systems scanners at a field strength of 3 Tesla, and each site followed its own routine clinical MRI protocol, resulting in some heterogeneity in image resolution and contrast. Site A contributed data from a range of scanner models across the three vendors: Siemens (Skyra, Verio, Prisma, Prisma_fit, TrioTim), Philips (Achieva, Achieva dStream, Intera, Ingenia), and GE Medical Systems (Signa HDxt, Discovery MR750, SIGNA Premier). Site B provided data acquired on Siemens Skyra and Skyra Fit scanners, while Site C used the Siemens Skyra Fit. Table 1 summarizes dataset characteristics across sites. The diversity of imaging sources and clinical presentations should reduce site-specific biases and enhance generalizability.

**Table 1:** Summary of site-specific data including number of cases, scanner vendors, and lesion characteristics.

Site	Train/Validation cases	Test cases	Scanner vendors	Median Lesion volume (cc)	Median Lesion count
Site A	105	44	Siemens, Philips, GE	4.54 [2.35–9.36]	44 [25–67]
Site B	247	78	Siemens	4.90 [1.53–13.81]	33 [19–55]
Site C	28	10	Siemens	2.48 [0.58–3.91]	27 [8–65]

To ensure data consistency, T1-weighted images were registered to their corresponding FLAIR sequences, and automated quality control was applied to identify potential image quality issues. This diverse dataset, representing multiple sites with varying imaging protocols, was used to assess the federated approach under realistic conditions of inter-site heterogeneity.

### 2.3 Preprocessing for image standardization

A standardized automated preprocessing pipeline was applied to ensure data consistency across all sites. This process included automated quality control procedures assessing key image properties. Signal-to-noise ratios (SNR) were computed in modality-specific anatomical regions to estimate overall image quality. T1w SNR was calculated in the brain parenchyma, while FLAIR SNR was calculated in the cerebrospinal fluid. Artifact presence was estimated using the MAI-Lab sorting and artifacts detection tool (17), and cropping was detected by evaluating brain coverage across anatomical boundaries. Voxel dimensions were validated against the expected isotropic resolution (1.0 ± 0.1 mm), and inter-modality brain mask volume similarity was assessed to detect major discrepancies or modality-specific artifacts. All MRI data were reoriented to a standardized axial orientation to ensure uniform spatial alignment. T1-weighted images were registered to their corresponding FLAIR images, correcting for positional misalignment. These preprocessing steps were performed locally at each site using Kaapana and integrated into the federated learning workflow, ensuring uniformity in the input data across sites for subsequent model training.

### 2.4 Model selection and training

We selected nnU-Net for its robust performance across diverse medical segmentation tasks, offering automatic adaptation and competitive results without manual customization (6). nnU-Net handles preprocessing, architecture selection, and postprocessing, reducing the need for extensive manual intervention. It is also well-suited for 3D multi-modal input, automatically configuring an appropriate 3D U-Net architecture based on input image dimensions and hardware constraints.

Local training at each site adhered to the standard nnU-Net training configuration and hyperparameters, with configurable values set to a learning rate of 0.01, weight decay of 3 × 10^−5^, 250 training batches per epoch, and 33% foreground oversampling. The model used the standard Dice loss combined with cross-entropy, as provided by the default configuration of nnU-Net. We conducted 50 rounds of federated training, with each round corresponding to one local epoch at each site. Limiting local training to a single epoch helped prevent models from overfitting to local data and drifting from the global objective. Training progress was monitored by tracking site-level training and validation losses after each federated round to ensure stability and detect potential divergence. After each round, the server aggregated client weight updates using the FedAvg algorithm to generate a new global model, which was then redistributed to all sites.

The final global model obtained after 50 rounds of federated training was evaluated independently at each site using its respective held-out test set. Model evaluation at the three participating sites included both quantitative metrics such as Dice score, sensitivity, and precision, as well as a qualitative review by a neuroradiologist to assess overall performance, including true positive detection and tendencies to miss lesions across anatomical regions. To benchmark against a non-federated scenario, we trained and tested a baseline nnU-Net model locally using our site’s data. This enabled a comparison between the performance of the federated model and the locally trained model, both evaluated on the same test set from our institution.

### 2.5 Privacy and security considerations

Patient privacy was a core requirement of our FL framework, which inherently avoids sharing raw imaging data. All images were anonymized at their source by removing identifying metadata (e.g., DICOM headers), ensuring that no personally identifiable information was accessible. Federated training was conducted within protected compute environments, with each site’s data remaining on secure local infrastructure. Our configuration follows enterprise-grade governance principles, ensuring that each client site retains full control over which algorithms are executed on its data. This level of control allows individual node administrators to prevent the execution of unauthorized or potentially malicious code, thereby strengthening overall system security.

As described in the Architecture section, only sites that received a secret, unique token were allowed to contribute to the central model updates, thus limiting potential poisoning attacks. Since communications between sites and the central server were TLS-encrypted, and additionally encrypted at the sites with a symmetric key shared within the token, the risk of an adversarial attack was minimal. The central cloud-based environment employed AWS Well-Architected Framework mechanisms, with access to the Federated Orchestrator restricted to a predefined IP range. This setup limited the marginal risk of reconstruction or inference attacks and allowed the use of original parameters and weights from individual nodes.

While FL reduces data privacy risks by design, it is not entirely immune to threats such as model inversion or membership inference attacks. To mitigate these risks, we adopted strict security principles integrated directly into the framework. Execution of any machine learning code or federated learning configuration requires explicit approval from each participating site. Comprehensive encryption and tightly controlled access to both site and central nodes further minimize the risk of sensitive data leakage or attacks by unauthorized, potentially malicious actors.

From a regulatory standpoint, this study adhered to data protection laws. Since only model parameters and not raw data were exchanged, each institution maintained full control over its data. Our framework serves as a starting point for multi-center collaborations, promoting secure AI development in medical imaging.

## 3 Results

### 3.1 Quantitative analysis

**Figure 2:**
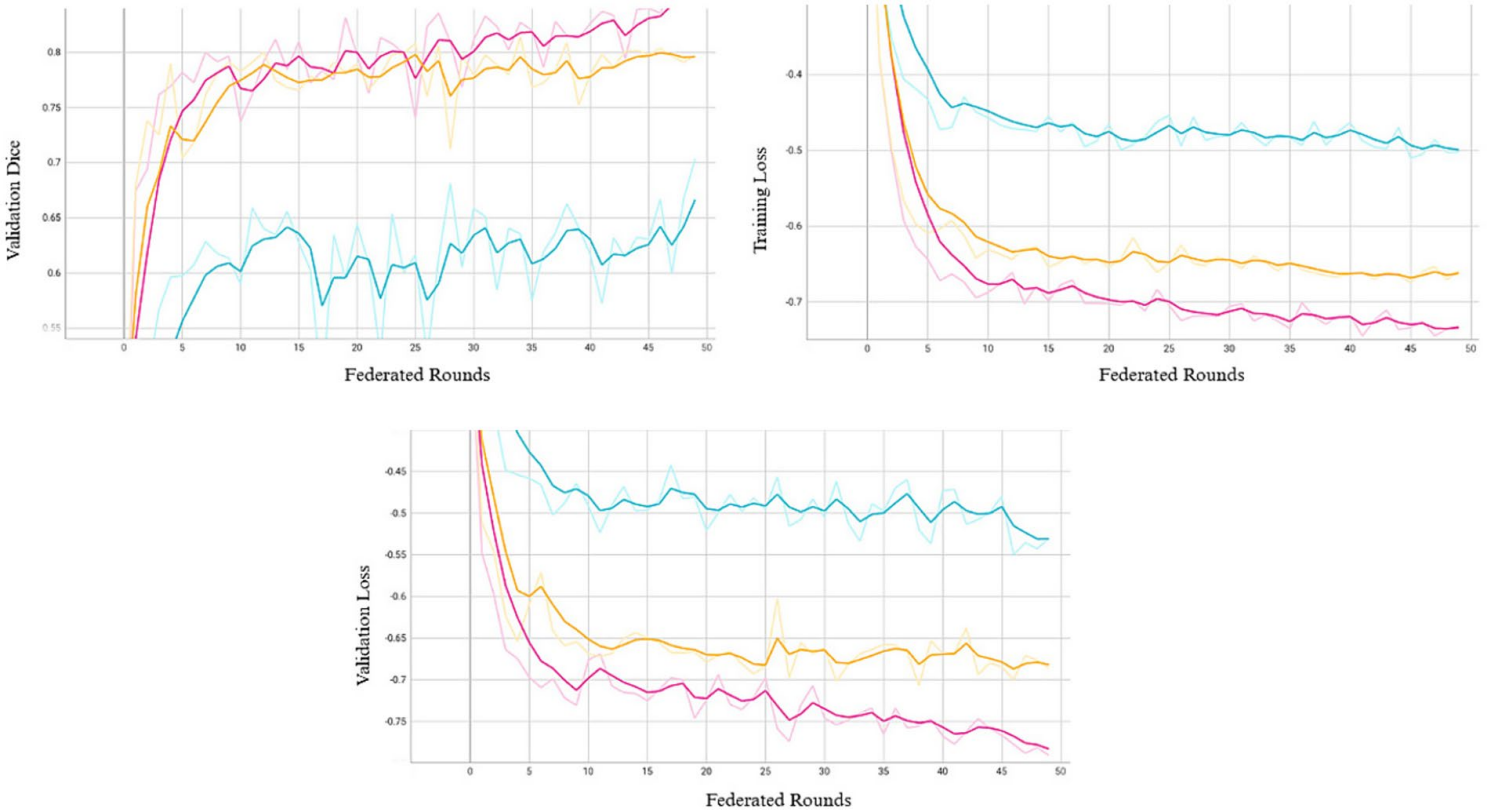
Validation Dice (top left), training loss (top right), and validation loss (bottom) across 50 federated rounds. Each curve represents one of the three participating sites (Site A: pink, Site B: orange, Site C: blue).

We conducted 50 rounds of federated training, with each round corresponding to one local epoch per site. In our setting, preliminary experiments with additional local epochs per round resulted in abrupt performance degradation, which may reflect FedAvg’s sensitivity to data heterogeneity (18). This aligns with observations in the literature where non-IID data or class imbalances can cause gradient misalignment, driving local models away from the global objective (19). Model performance over 50 federated rounds is shown in Figure 2. Most reductions in training and validation losses occurred within the first 10–15 rounds, after which learning progressed more gradually. As the system retains only model weights from the final training round, we adopted a fixed training schedule rather than an adaptive early stopping strategy based on convergence. Extended training revealed that additional rounds improved performance at certain sites, while others experienced a decline, potentially due to model drift or overfitting to dominant patterns. Although 50 rounds may not represent the global optimum, this configuration provided a balanced trade-off across all participating sites.

To assess model performance, we compared a locally trained model to its federated counterpart using a held-out test set of 44 MRI cases from our site. Both models were trained using the same hyperparameters and configuration to ensure a fair comparison. A local nnU-Net model trained for 50 epochs using only our site’s training set achieved a mean Dice score of 0.88 ± 0.04, sensitivity of 0.85 ± 0.05, and precision of 0.90 ± 0.05 on our site’s test set. In comparison, the federated model, trained for 50 rounds with one local epoch per round across the three sites, achieved a mean Dice score of 0.80 ± 0.07 on the same test set. While the federated model showed a lower Dice score, it demonstrated higher sensitivity (0.89 ± 0.07 vs. 0.85), indicating improved lesion detection, albeit with reduced precision. One-sided Wilcoxon tests indicated that the local model had significantly higher Dice and precision (*p* = 5.7 × 10^−14^ for both). In contrast, the federated model showed significantly higher sensitivity based on a one-sided paired t-test (*p* = 2.4 × 10^−7^). In a clinical context, higher sensitivity is valuable for minimizing the risk of missed lesions; however, the corresponding decrease in precision reflects a higher rate of false positives, which may result in unwarranted diagnostic procedures, increased clinician workload, and patient distress.

To evaluate the cross-site generalizability of the federated model, we evaluated it on held-out test sets from the other two participating sites, comprising 78 and 10 cases, where it achieved mean Dice scores of 0.71 ± 0.15 and 0.66 ± 0.16, respectively. These results are summarized in Table 2. A Kruskal-Wallis test across all sites showed significant site-dependent variability in Dice scores (*p* = 3.05 × 10^−5^). Given the limited test sample size at Site C, we further conducted a two-sided Mann–Whitney U test between Site A and Site B, which also indicated a statistically significant difference in Dice scores between the two sites (*p* = 3 × 10^−5^).

**Table 2:** Federated model performance on the test set from each participating site.

Site	Dice Score	Sensitivity	Precision
Site A	0.80 ± 0.07	0.89 ± 0.07	0.74 ± 0.11
Site B	0.71 ± 0.15	0.74 ± 0.11	0.70 ± 0.17
Site C	0.66 ± 0.16	0.64 ± 0.19	0.74 ± 0.23

Metrics are reported as mean ± standard deviation for Dice score, sensitivity, and precision, highlighting inter-site variability in segmentation accuracy.

**Figure 3:**
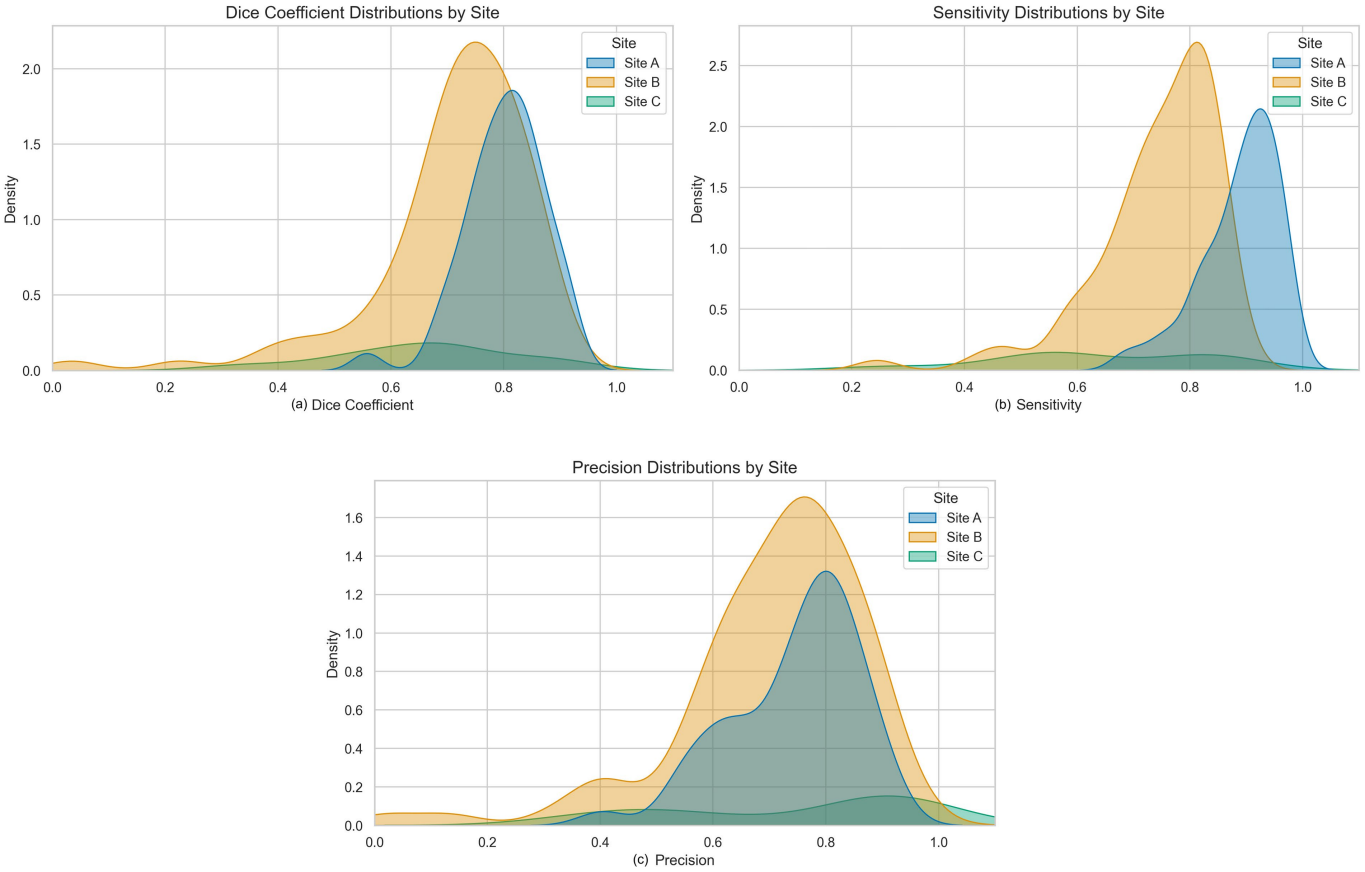
Density plots of segmentation metrics across sites. The plots show the distribution of **(a)** Dice score, **(b)** sensitivity, and **(c)** precision for Site A, Site B, and Site C, reflecting inter-site variability in segmentation performance.

Figure 3 presents the distribution of performance metrics for each site, with Sites A and B showing relatively more consistent distributions and Site C exhibiting broader variability, reflecting inter-site differences in model generalization. Although performance varied, likely due to differences in imaging protocols, scanner types, or annotation standards, the model maintained moderate segmentation performance across diverse clinical environments without access to raw patient data. Importantly, federated training does not preclude subsequent site-level adaptation. Fine-tuning the global model on local data can help capture site-specific patterns, offering a balanced approach that preserves the robustness gained from diverse data while recovering the precision of locally optimized models.

### 3.2 Qualitative assessment

To complement the quantitative evaluation, a qualitative radiological assessment was conducted to examine the alignment between visual observations and metric-based performance. A board-certified neuroradiologist and MS expert assessed aspects not fully captured by global quantitative metrics, such as pathological plausibility (e.g., false negatives and false positives), anatomical consistency (e.g., periventricular, subcortical, and other region-specific biases), and morphological correctness (e.g., small versus large lesions). The expert reviewed lesion masks generated by (1) the federated model trained across all sites and (2) a model trained solely on local data from our site. As in the quantitative evaluation, the comparison was performed on outputs generated from the held-out test set at our site, with the models’ outputs reviewed side by side to identify clinically meaningful differences in segmentation behavior.

**Figure 4:**
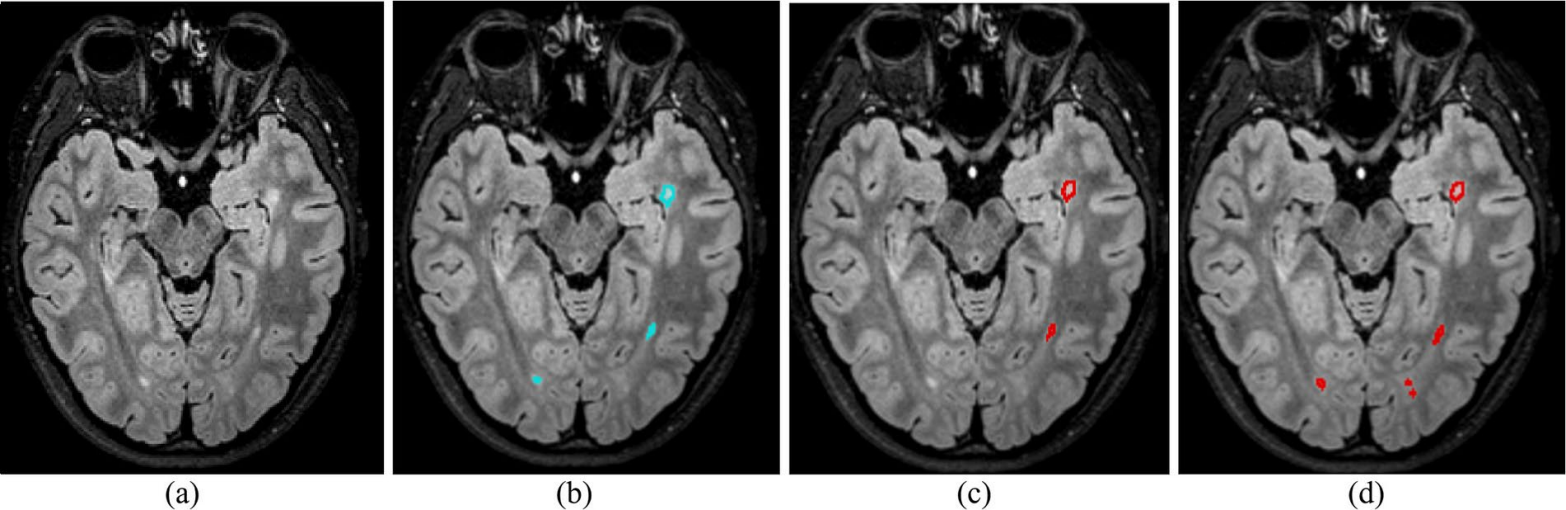
Comparative visualization of lesion segmentation between training paradigms: **(a)** FLAIR MRI slice without annotations; **(b)** Ground truth manual segmentation; **(c)** Prediction from the local model (trained solely on our data); **(d)** Prediction from the federated model (trained across three sites). The federated model detects more lesions but also introduces additional false positives, reflecting the trade-off between sensitivity and precision.

Figure 4 presents a visual comparison on a FLAIR slice from our site’s test set, with model predicted segmentation masks overlaid on the image. The results highlight key differences between the models, with the federated model detecting more lesions, reflecting higher sensitivity, but also introducing more false positives. While further validation is warranted, these findings demonstrate the feasibility of federated learning for automated MS lesion segmentation, underscoring its potential for broader clinical application.

## 4 Discussion

This Proof of Concept study demonstrates the end-to-end technical feasibility of deploying federated learning as a scalable, privacy-preserving framework across clinical institutions, each with distinct privacy constraints, data governance policies, and technical environments. Our work addresses a gap often overlooked in simulated FL research by preserving full data governance at each site while supporting scalable algorithm integration and institutional participation. By integrating Kaapana and Apheris, our framework enables autonomous data curation and enforces consensus-based algorithm approval prior to execution at each site, enhancing both privacy and operational security. This design allows each institution to manage its own imaging workflows while safeguarding against unauthorized computation, making the approach particularly well-suited for sensitive clinical environments. This federated setup is inherently portable and supports scalable, efficient deployment. It can be extended to additional institutions by deploying a platform instance at each site with secure client-to-server communication. This modular architecture emphasizes flexibility, reproducibility, and compatibility with diverse governance policies, enabling broader future adoption.

Building on this infrastructure, we evaluated the federated model on the held-out test set from each participating site. For comparative analysis, we also compared its performance on our site’s held-out test set relative to a model trained and tested locally. Although the federated model showed a lower Dice score compared to the locally trained model at our site, it achieved higher recall, which may indicate improved lesion detection. This trade-off reflects a core challenge in FL as it requires balancing global generalization with site-specific optimization. The observed performance gap in Dice score likely stems from the federated model’s exposure to heterogeneous, non-IID data across institutions, which encourages learning generalized representations rather than overfitting to any specific site’s patterns. Federated models are optimized to perform robustly across diverse data distributions, enhancing sensitivity to subtle or atypical lesions that may be underrepresented in any single site’s dataset. However, this improvement in sensitivity was accompanied by reduced precision, as the federated model might not fully adapt to site-specific imaging features and annotation styles. This misalignment may cause the model to over-segment or misclassify challenging regions, resulting in an increased number of false positives. Additionally, while local training on homogeneous data can converge rapidly, federated learning may require more rounds to achieve comparable performance due to the challenges of learning from fragmented and non-IID data distributions.

Beyond performance trade-offs, our study highlights several practical challenges that are often overlooked in simulated FL settings. First, in synchronous FL workflows, training requires all sites to remain active; resource outages or downtime at any site can halt the entire federated round. Second, training local models for comparison with the federated model requires technical expertise at all participating sites, which may not always be readily available. In contrast, participation in federated training and quantitative evaluation of the federated model in our setup did not require machine learning expertise. Third, centralized baseline models trained on pooled multi-site data, which are commonly used as performance upper bounds for federated models, are often infeasible in real-world clinical settings due to data privacy regulations, as was the case in our study. These constraints underscore the gap between FL in theory and its real-world implementation.

It is also worth noting that the federated model in this Proof of Concept study was not intended to optimize performance, and thus was only trained on a relatively small dataset (380 cases), whereas many deep learning studies rely on datasets exceeding 1,000 cases (20) or even tens of thousands in population-scale initiatives like UK Biobank (e.g., 39,694 subjects (21)). While expanding to larger, more diverse cohorts is expected to improve generalizability, site-specific accuracy gains may require complementary strategies. For instance, fine-tuning the federated model on local data can improve local performance, but risks catastrophic forgetting, where local adaptation distorts generalizable representations learned during federated training, leading to degraded performance on external datasets. To address this, personalized FL strategies such as FedBN (22), which retains local batch normalization statistics to account for domain shifts, and Ditto (23), which optimizes a personalized objective while maintaining alignment with the global model, have shown promise in non-IID settings. Additionally, adaptive aggregation adjusts client contributions to better manage data skew, with methods like FedProx (24) introducing a proximal term to reduce client drift and improve convergence stability.

While this study demonstrates the technical feasibility of FL in real-world settings, future research should explore integrating adaptive aggregation, personalized FL strategies, and expanding datasets to further improve model performance in heterogeneous environments. Overall, these findings establish a starting point for adopting federated learning in clinical practice, with potential for future scaling to multi-modal and longitudinal MS studies.

## Software and resources

The federated learning infrastructure was implemented using the open-source Kaapana platform (https://github.com/kaapana/kaapana), with the nnU-Net training pipeline available at https://github.com/kaapana/kaapana/tree/develop/data-processing/processing-pipelines/nnunet. Additional computational governance capabilities were supported by Apheris (https://www.apheris.com), enabling secure collaboration across participating institutions.

## Data Availability

The data analyzed in this study is subject to the following licenses/restrictions: the datasets consist of anonymized clinical MRI data from a previous Roche-sponsored trial and observational data from two academic medical centers. All data remained local to each site and were not transferred or shared centrally, in accordance with data protection regulations. The datasets are not publicly available due to patient privacy considerations and institutional data protection agreements, and therefore are not included in the manuscript or supplementary files. Requests for further information regarding the data or methodology may be directed to the corresponding author, Sarah Hindawi (sarah.hindawi@roche.com).
